# SEM Image Processing Assisted by Deep Learning to Quantify Mesoporous γ-Alumina Spatial Heterogeneity and Its Predicted Impact on Mass Transfer

**DOI:** 10.1021/acs.jpcc.4c00323

**Published:** 2024-05-13

**Authors:** Aleksandra Głowska, Elsa Jolimaitre, Adam Hammoumi, Maxime Moreaud, Loïc Sorbier, Caroline de Faria Barros, Veronique Lefebvre, Marc-Olivier Coppens

**Affiliations:** † Centre for Nature Inspired Engineering and Department of Chemical Engineering, University College London, London WC1E 7JE, United Kingdom; ‡ IFP Energies Nouvelles, Rond-point de l’échangeur de Solaize, BP 3, Solaize 69360, France

## Abstract

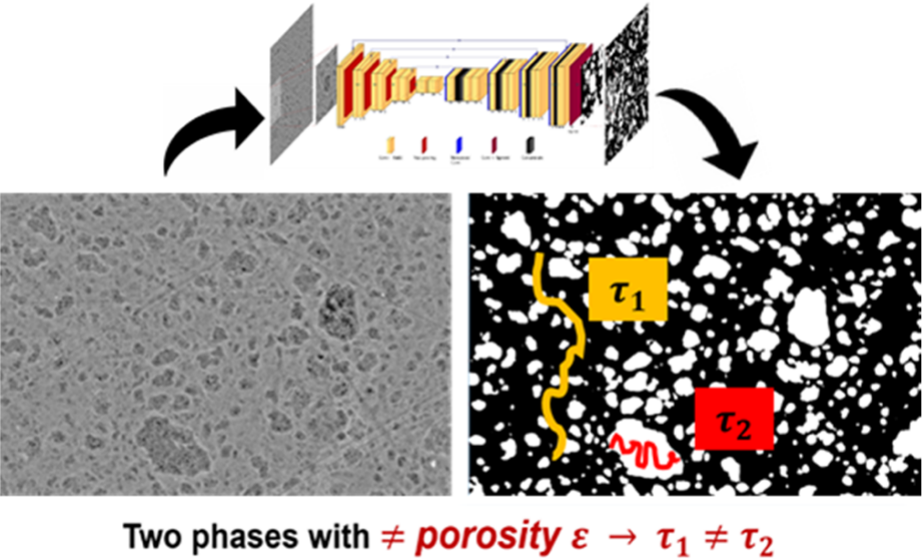

The pore network architecture of porous heterogeneous catalyst supports has a significant effect on the kinetics of mass transfer occurring within them. Therefore, characterizing and understanding structure–transport relationships is essential to guide new designs of heterogeneous catalysts with higher activity and selectivity and superior resistance to deactivation. This study combines classical characterization via N_2_ adsorption and desorption and mercury porosimetry with advanced scanning electron microscopy (SEM) imaging and processing approaches to quantify the spatial heterogeneity of γ-alumina (γ-Al_2_O_3_), a catalyst support of great industrial relevance. Based on this, a model is proposed for the spatial organization of γ-Al_2_O_3_, containing alumina inclusions of different porosities with respect to the alumina matrix. Using original, advanced SEM image analysis techniques, including deep learning semantic segmentation and porosity measurement under gray-level calibration, the inclusion volume fraction and interphase porosity difference were identified and quantified as the key parameters that served as input for effective tortuosity factor predictions using effective medium theory (EMT)-based models. For the studied aluminas, spatial porosity heterogeneity impact on the effective tortuosity factor was found to be negligible, yet it was proven to become significant for an inclusion content of at least 30% and an interphase porosity difference of over 20%. The proposed methodology based on machine-learning-supported image analysis, in conjunction with other analytical techniques, is a general platform that should have a broader impact on porous materials characterization.

## Introduction

γ-Alumina (γ-Al_2_O_3_) has found widespread application as a catalyst or catalyst support in the chemical, refinery, and automotive industries over the last 50 years,^1,2^ yet the prediction of its mass transfer properties still represents a major challenge due to the high complexity of its porous structure. Heterogeneous catalytic processes are often diffusion-limited at the catalyst pore network level, and, therefore, a comprehensive understanding of the porous structure–transport relationship is crucial to control diffusion in these processes and to optimize the performance of heterogeneous catalysts.

Previous studies devoted to textural characterization of γ-alumina have highlighted two principal aspects to which its structural complexity can be traced. First, the porous structure results from several levels of aggregation of elementary boehmite nanoparticles (∼5 nm), being the precursor of γ-alumina. The number of aggregation levels, as well as the size and porosity of each level of aggregates, strictly depend on the applied synthesis conditions during the boehmite precipitation, but also peptization and neutralization stages through the addition of acids and bases, respectively.^3−6^ In the study of Forman et al.,^7^ the formation of two porosity levels was reported, referred to as intra- and interaggregate porosity. Kolitchev et al.^8^ describe that the first porosity level (i.e., intra-aggregate porosity) is created between the alumina nanoparticles forming an aggregate, followed by a second, larger porosity level, which develops between the aggregates (i.e., interaggregate porosity) when they are compressed to form the catalyst support. Therefore, the pore network of γ-alumina is characterized by a complex hierarchical organization, ranging from the nanometer to the millimeter scale.

The second aspect, which is the subject of this study, involves spatial heterogeneity in the form of aggregates of different sizes and densities, which can be present across different levels of pore network organization. Already at the nanoscale, Wang et al.^9^ observed changes in local alignment of alumina nanoparticles, and, therefore, in the stacking of these nanoparticles forming the aggregates. Furthermore, γ-alumina’s spatial heterogeneity is often reported at the microscale, in the form of alumina inclusions embedded in an alumina matrix with a remarkable density difference between both phases observable on scanning electron microscopy (SEM) images.^7,10,11^

These heterogeneities in the form of inclusions can influence the mechanical and transport properties of porous materials. Since alumina inclusions are easily observable by SEM, their quantification can be performed via SEM image processing methods to evaluate their impact on the mentioned properties. Such quantification of alumina inclusions has been reported by Dengiz et al.,^12^ where a quantitative analysis of dense heterogeneities in partially sintered alumina was performed to evaluate their effect on mechanical strength. In this study, automated SEM image analysis with an adaptive thresholding algorithm^10^ was applied for the quantification of inclusion area fraction and size distribution.

SEM image analysis approaches are often applied to evaluate different pore space properties of porous solids. In the context of aluminic materials, Raimundo et al.^13^ processed SEM images by using a gray-level thresholding method to obtain binarized images, which served to calculate the minimum, maximum, and average pore radius distribution of anodic porous alumina structures. This analysis enabled them to extract the porosity by computing the area occupied by pores. Choudhari et al.^14^ also analyzed SEM images of nanoporous anodic alumina membranes by applying a segmentation approach based on the active contour model^15^ to obtain binarized images for determination of the average interpore distance and size distribution, porous area fraction, and pore circularity.

Mass transfer in nanoporous materials has been reviewed by Kärger et al.^16,17^ both as regards experimental measurement techniques and theoretical modeling, but studies on the impact of spatial heterogeneity on mass transfer are rather scarce. Using a combination of nuclear magnetic resonance (NMR) techniques, previous studies by Rigby et al.^18^ and Hollewand and Gladden^19,20^ have mapped porosity and mean pore radius variations of alumina pellets and measured the effective diffusion coefficient of water in the pore network. The impact of macroscale heterogeneity on mass transport was qualitatively shown in these pioneering studies. However, the pixel size was too large on the order of several dozens of micrometer so that subtle mesoporous scale heterogeneities could not be detected. Moreover, without implementation of image analysis techniques, a robust quantitative analysis could not be achieved.

Tariq et al.^21,22^ reconstructed in three-dimensions (3D) the pore network of alumina using multiscale tomography. Images were analyzed based on grayscale levels. Each pixel was attributed to either a void or an alumina matrix, and the pore network was reconstructed as the sum of all void pixels. Hence, only voids whose size represents at least one pixel were detected, meaning that all pores under 10 nm, representing more than 50% of the total porosity, were not considered. A similar methodology (microtomography coupled with grayscale image analysis) was implemented by Ruffino et al.^23^ with comparable conclusions: only the macropores were considered for the network 3D reconstruction.

Recently, a first study of the heterogeneity of γ-alumina porosity to involve digital image processing was carried out by Sorbier et al.^11^ The method required specific sample preparation, involving the impregnation of γ-alumina catalyst supports with a resin and performing SEM imaging in backscattered electron mode on their cross sections. This allowed the calibration of image gray levels with those of the pure resin and bulk alumina, which enabled the automated measurement of local porosity from SEM images. The study provided important insight into the impact of the synthesis conditions on γ-alumina porosity. Interestingly, the higher porosity values measured by SEM for samples produced using increased kneading energy were explained by the destruction of dense alumina inclusions, which were transformed into a less dense phase with a higher porosity, represented by the alumina matrix. This implies a direct impact of alumina inclusions on mean support porosity so that mass transfer could also be influenced by the presence of inclusions.

For heterogeneous two-component materials, the literature contains a range of basic, two-phase analytical models to predict their effective physical properties, which can also be used to predict mass transfer.^24^ These models require the knowledge of local parameters, including the volume fraction and porosity of each phase, which could be determined by using appropriate SEM image processing approaches for image segmentation or binarization, yet this remains extremely challenging for γ-alumina supports due to the textural particularity of its components, which are difficult to define with standard statistical descriptors.

To the best of our knowledge, this work presents a first, detailed quantitative study of γ-alumina’s spatial mesoporous heterogeneity with evaluation of how this could impact mass transfer. The local porosity measurement method proposed by Sorbier et al.,^11^ coupled with image segmentation and analysis approaches, was applied to γ-alumina supports with two distinguishable phases, in order to evaluate the local properties of each of the components relevant for mass transfer predictions, including their volume fractions and porosities. The difficulties encountered during the SEM image segmentation of some aluminas with particular textural complexity are discussed, which led to the development of a new methodology based on deep learning semantic segmentation employed in this study. Finally, local quantification results from SEM are used as input for the Bruggeman-type effective medium theory (EMT)^25^ and reciprocity^26,27^ models to predict the effective tortuosity factor from the contribution of both phases. The impact of alumina heterogeneities is assessed in terms of the inclusion content and porosity heterogeneity.

## Methodology

### Materials Studied

Two boehmite-derived γ-alumina catalyst supports, termed A and B, were provided by IFPEN for the purpose of this study. Both supports were synthesized via aluminum salt coprecipitation in an aqueous solution. The precipitated boehmite was filtered and rinsed with water to remove impurities for the acidic and basic precursors and then dried to evaporate the solvent. The shaping procedure involved the transition from a boehmite powder to cylindrical support extrudates with a diameter of 1.6 mm through kneading (with nitric acid and ammonia solutions) and extrusion. Finally, the transformation of boehmite to γ-alumina was performed through calcination at 540 °C. For both aluminas, the synthesis conditions were modified to obtain supports with very different diffusional and textural properties. These different textural properties require the implementation of different image treatment methodologies for each sample.

### Characterization Techniques

#### Nitrogen Physisorption and Helium Pycnometry

Each support’s textural properties, such as the specific surface area, *S*_BET_, and specific pore volume, *V*_pore_, were determined from the N_2_ adsorption and desorption isotherms measured on a 3Flex instrument (Micromeritics) after pretreatment under secondary vacuum (10^–5^ mbar) at 350 °C for 3 h to remove physisorbed and chemisorbed water. These conditions are more stringent than those prescribed by the D3663 ASTM method (300 °C, 3h with pressure lower than 0.13 Pa). During isotherm acquisition, equilibration is tested over periods of 10 s. Equilibration is considered to be reached if the pressure change during the current 10 s period is less than 0.01% of the mean pressure during this period. The expected relative uncertainty in *V*_pore_ is 3%. The pore size distribution (PSD) was evaluated from the N_2_ adsorption and desorption isotherm branches using an NLDFT model for slit pores (77 K N_2_ DFT Model, MicroActive software) and the BJH method. Structural density, ρ_s_, was measured by helium pycnometry on an AccuPyc 1340 instrument (Micromeritics). This enabled the evaluation of total porosity, ε_N_2__, using the following equation
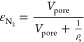
1

#### Mercury Intrusion Porosimetry

To account for the possible presence of macropores, the alumina supports were further characterized by mercury intrusion porosimetry (MIP) on an AutoPore IV instrument (Micromeritics). Prior to analysis, both aluminas were subjected to a pretreatment at 250 °C for 2 h to ensure equilibration.^28^ The PSD was evaluated from the mercury intrusion process to compare with the N_2_ desorption PSD. The grain density, ρ_g_, was measured at an intrusion pressure of 0.2 MPa, which corresponds to the filling of only the intergrain porosity with an expected relative uncertainty of 5%. The total porosity from mercury intrusion, ε_MIP_, was then determined as
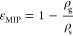
2

#### SEM Imaging in the Backscattered Electrons Mode for Alumina Heterogeneity and Porosity Quantification

**Figure 1:**
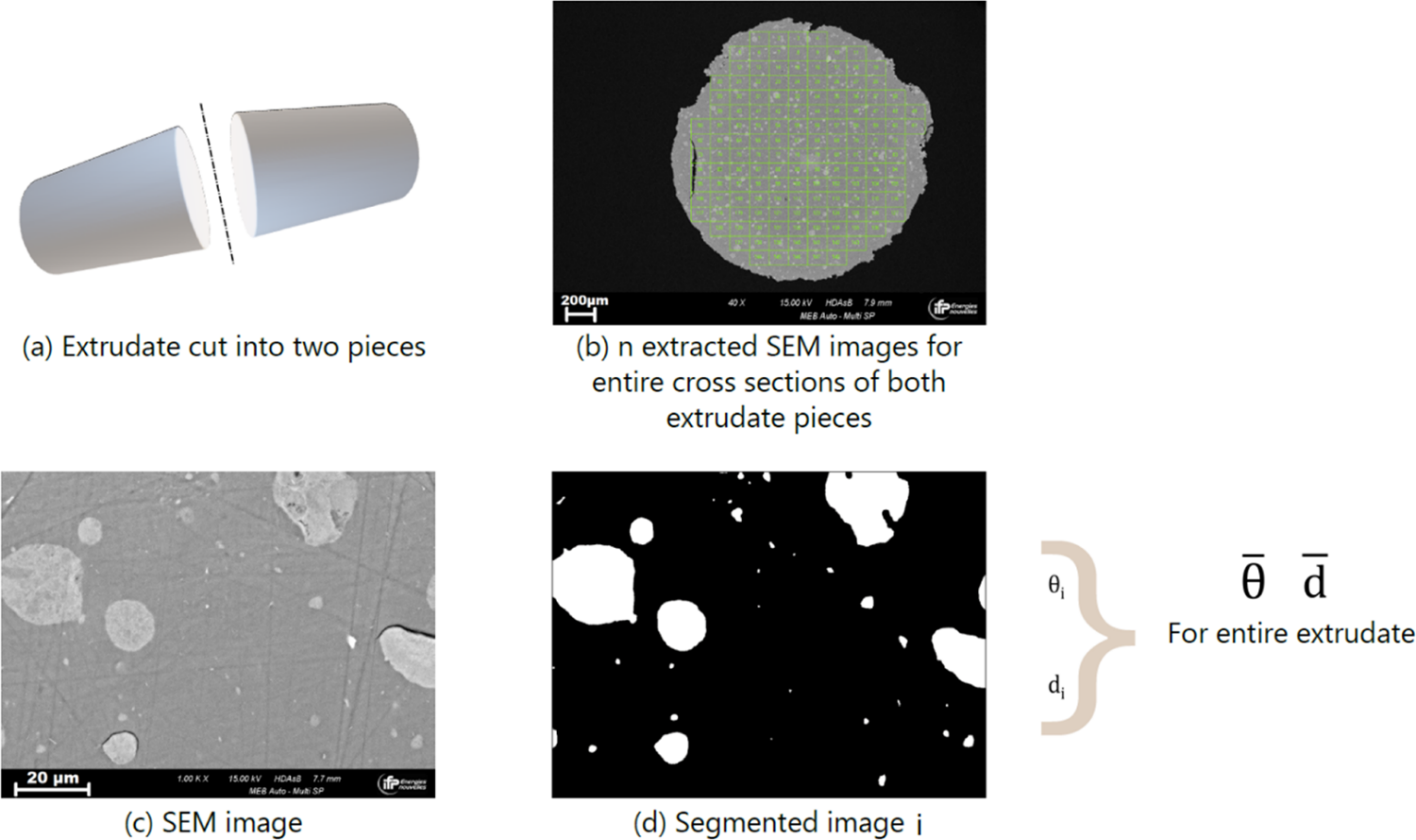
SEM imaging and processing methodology for alumina heterogeneity quantification: (a) extrudate cut into two pieces for analysis, (b) alumina B extrudate cross section digitally divided into individual images of 1024 × 768 pixels, (c) SEM image to be processed, and (d) segmented SEM image obtained via gray-level segmentation to extract inclusion area fraction θ_*i*_ and diameter *d*_*i*_. Total inclusion area fraction θ̅ and average inclusion diameter *d̅* are evaluated based on the total number of images *n* acquired for the cross sections of both extrudate pieces.

For each support, one extrudate was taken and dried overnight at 50 °C. Extrudates were then cut into two sections, as shown in Figure 1a, and each section was impregnated under pressure in a mixture of liquid methyl methacrylate (MMA, Sigma-Aldrich) and azo-bis-isobutyronitrile (AZDN, Sigma-Aldrich), used as a radical polymerization initiator. As a result of in situ polymerization of MMA to poly-MMA in the pores of alumina, a uniform impregnation of the entire support porosity is obtained, which has been confirmed by Sorbier et al.^11^ The extrudate sections were then mechanically polished under water to #4000 grit (SiC paper) using an automatic Buehler AutoMet 250 polisher. This preparation ensures sufficient resolution for spatial heterogeneity imaging. The sample preparation methodology and polymerization conditions have been fully described by Sorbier et al.^11^ Prior to SEM analysis, all samples were metalized with carbon.

The resulting cylindrical stubs (i.e., two for each support, containing individual extrudate sections) were positioned in a sample holder and imaged under a Zeiss SUPRA 40 scanning electron microscope using an HDAsB detector at 15 kV and a working distance of 8 mm. By using dedicated software developed via the Zeiss application programming interface (API), the entire transverse cross section of the extrudate was digitally divided into *n* rectangular zones with dimensions of 1024 × 768 pixels (Figure 1b) and automatic image acquisition was performed for all zones, while keeping the same imaging parameters as described above. All images were acquired at a magnification of ×1000 in 8 bits depth (example in Figure 1c).

For the porosity quantification, the same polished sections of alumina extrudates embedded in the PMMA resin were used. For each alumina support, 6 images were acquired for predefined zones of each extrudate section, yielding a total of *m* = 12 images for each sample, taken at a magnification of ×1000, optimal working distance of 4.2 mm, and resolution of 1024 × 768.

### SEM Image Processing for Spatial Heterogeneity Quantification

A semantic segmentation operation was carried out on alumina SEM images to distinguish and quantify the different levels of heterogeneity in the material, corresponding to the alumina inclusions and the matrix. Semantic segmentation is a binary classification task, where each pixel is assigned a value indicating its attribution to either the inclusions or the matrix (Figure 1d). The classification rule is determined by analyzing the pixel values of each level of heterogeneity. In the case of low contrast and complex textural variations, classical approaches to image processing have proven to be ineffective. One way to overcome this limitation is to rely on the deep learning paradigm using a convolutional neural network approach.

In recent years, deep convolutional neural networks (DCNNs) have started to outperform several classical image processing methods in handling problems such as semantic segmentation, image classification, and other ones.^29^ The two aluminas studied represent different types of supports with varying degrees of textural complexity and gray-level contrast between both components (i.e., inclusions and matrix) and, therefore, require two distinct semantic segmentation strategies. For Alumina B, a set of classical image processing operations were used to segment the SEM images, whereas Alumina A required a convolutional neural network approach.

#### Semantic Segmentation of Alumina B

**Figure 2:**
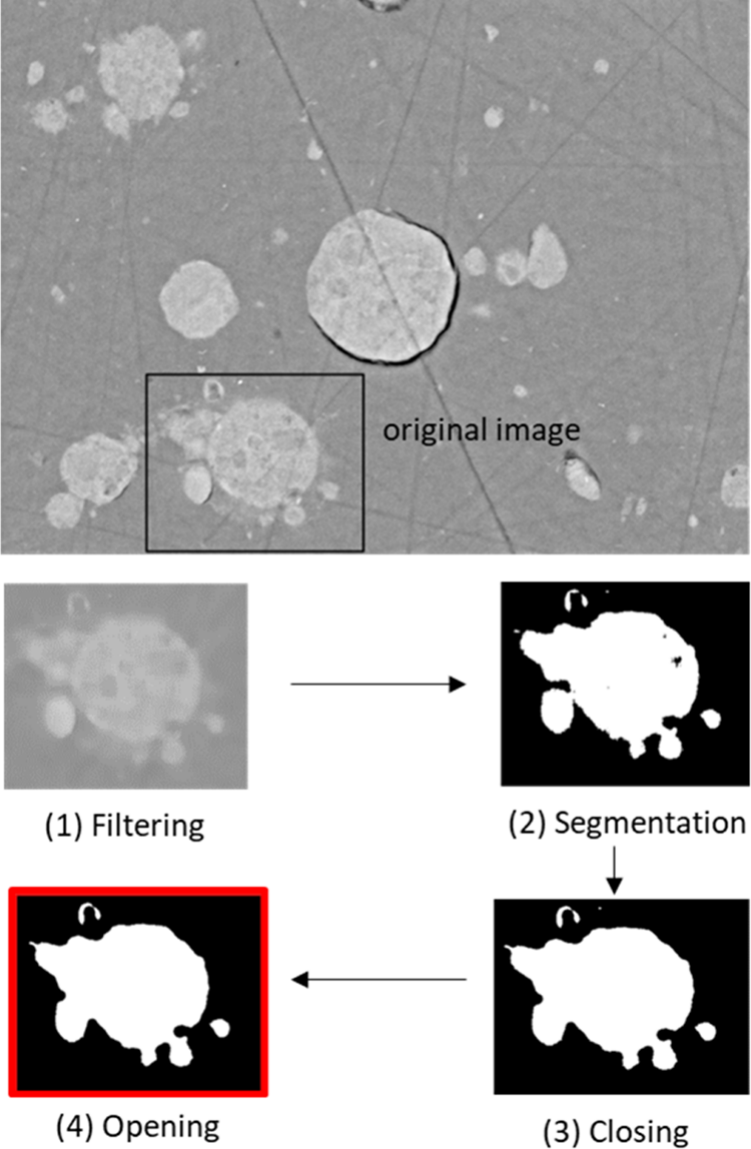
Slice of support B and related operations for semantic segmentation: (1) flowing bilateral filter, (2) Otsu’s method segmentation, (3) morphological closing, and (4) morphological opening.

Support B has little textural variation, and the contrast between the levels of heterogeneity is significant. In this case, it is possible to segment the image using a set of nonlinear successive transformations, as shown in Figure 2. First, a noise reduction operation is applied by using a bilateral filter. The latter replaces the intensity of each pixel by a weighted average of the intensity values of the neighboring pixels, reducing noise in the homogeneous region while preserving edges of the heterogeneity. The flowing version of the bilateral filter proposed by Moreaud and Cokelaer^30^ was used. They proposed a way of calculating the tonal weighting coefficients to reduce the halo artifacts produced by an ordinary bilateral filter. In the second step, a segmentation operation is performed to separate the material heterogeneities into two classes, namely, inclusions and the matrix. This operation is based on Otsu’s method,^31^ allowing us to calculate the optimal threshold separating the two classes on the histogram of pixel intensities by minimizing their intraclass variance. Morphological operations^32^ are then applied, in particular, a closing morphological operation with a disk structuring element with a radius of 6 pixels to fill narrow regions and small holes of the same segment. That is, the closing operation serves as a corrector for some of the usual artifacts of the segmentation operation, such as the division of a set of pixels of one class into multiple small sets. Finally, a morphological opening operation was used to remove small sets of pixels considered as residual noise (with an area smaller than 100 pixels).

#### Semantic Segmentation of Alumina A

**Figure 3:**
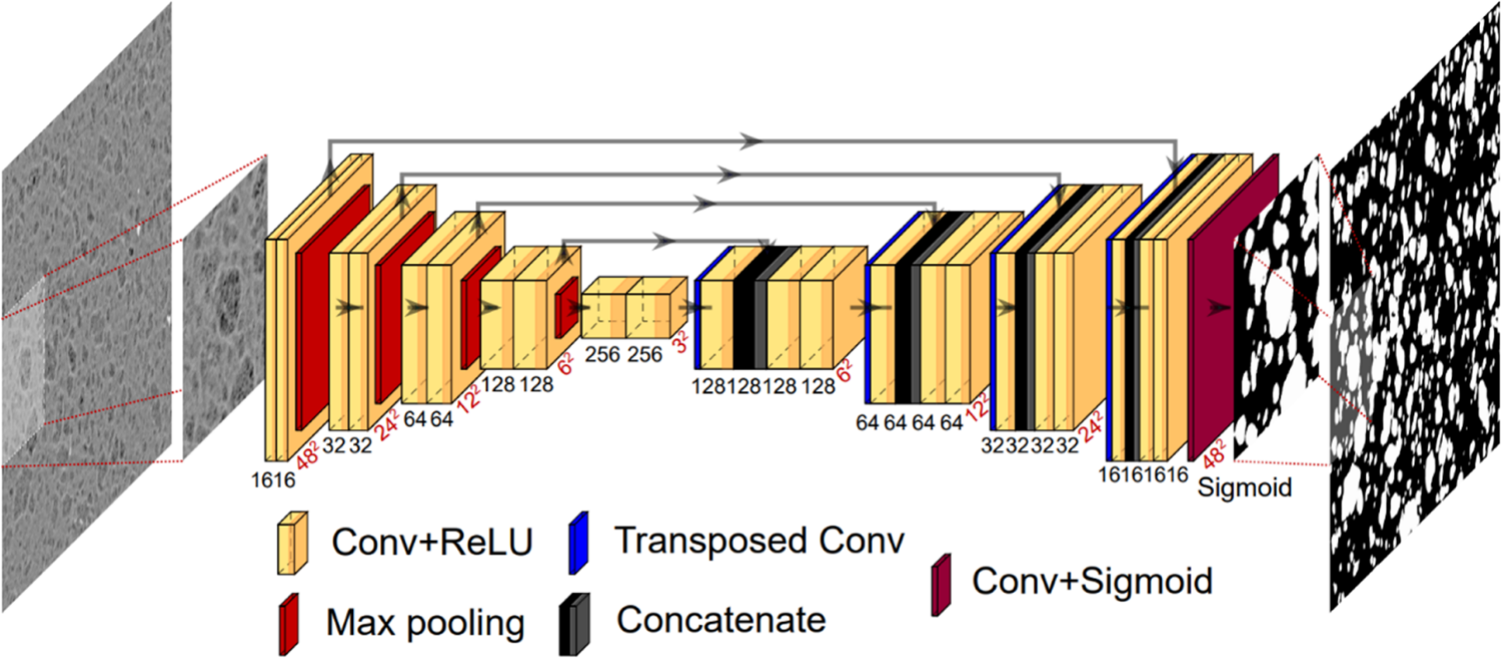
U-Net architecture characterized by a contraction path (left) and an expansion path (right). Operations are convolution, transposed convolution, maximum pooling, and concatenation. Two activation functions are used: ReLU and sigmoid. The input of the network is a set of patches of size 48 × 48 extracted from slices of the original alumina support A. Each output image is reconstructed by a stochastic assembly of predicted patches of its corresponding input image.

Gauging the data representation characterized by complex textural variations exhibited by support A is more difficult and requires a deep learning approach. Indeed, the contrast between the matrix and the inclusions is not high enough to use the classical gray-level segmentation described before (see Figure S4 in the Supporting Information: the lack of a clear and distinct contrast in grayscale between the matrix and the inclusions complicates their differentiation via grayscale level segmentation). The architecture of autoencoders as neural networks allows us to learn about discriminative features by reducing the dimension of the initial data and calculating feature maps along a compression path. Down-sampling the input representation (slice of support A) can be done by a sample-based discretization process, such as Max pooling, whereas extracting abstract representations in the form of feature maps is done by convolution matrices. Subsequently, an expansion path allows the reconstruction of the output image by increasing the resolution of the compressed data through up-sampling operators, such as transposed convolution matrices. The network in this work is based on U-Net,^33^ which is a popular convolutional neural network. In addition to the former steps involved in a classical autoencoder architecture, the latter supplements the network with a concatenation procedure allowing regaining of the spatial information lost by transferring feature maps to the expansion path through layer-by-layer correspondence. Afterward, a (1 × 1) convolutional layer followed by an appropriate activation function yields a precise reconstruction of the output image from the information collected beforehand. A complete description of the network is provided in Figure 3.

It is often necessary to have a large learning data set to train a neural network. However, the process of preparing training images can be slow and fastidious in many fields. In our case, 30 images of support A were manually segmented with respect to the two components (i.e., inclusions and matrix) to build the training data set. Since this number of samples is insufficient to train the model properly, a patch training strategy introduced by Hammoumi et al.^34^ was used. This approach consists of decomposing an image into several regions on which the training will be based. For instance, 1291 half-overlapping patches of size 48 × 48 can be generated for each image of size 1024 × 768. At the inference time, the predicted segmented image is fully assembled by a stochastic process drawing the patches with random coordinates. This stochastic assembly of patches, introduced as a stratified sampling strategy by Hammoumi et al.,^34^ allows to avoid edge effects at the border of patches when they are regularly distributed. The U-Net architecture adapted to our data set is shown in Figure 3.

#### Quantification of Spatial Heterogeneity

The quantification of spatial heterogeneity for both γ-aluminas was performed on binary images obtained through the semantic segmentation procedure, on which the heterogeneities are visible in white and separated from the alumina matrix in black (example in Figure 1d). For each image *i*, the surface fraction of heterogeneities θ_*i*_ was determined by counting the fraction of white pixels in the image. The average surface fraction of alumina heterogeneities θ̅ was obtained by averaging the θ_*i*_ for both aluminas from the total number of binary images obtained for the entire cross-sectional surface of each section (Figure 1b). The spatial distribution of alumina heterogeneities throughout the support extrudate’s transverse cross section was examined.

The diameter of alumina heterogeneities *d* was defined as the largest distance between two pixels at the contours of a connected component of the segmented images representing the heterogeneities, and the physical size was obtained by multiplying the distance in pixels by the pixel size of 0.11165 μm. The number frequency size distribution of alumina heterogeneities in both supports was then determined based on the entire binary image set obtained for each extrudate section. Prior to size evaluation, alumina heterogeneities cropped by image contours were digitally removed to avoid size misestimation. Additionally, all heterogeneities with a surface area inferior to 100 pixels^2^ were excluded from both the surface fraction and size quantification due to the lack of such small objects in the training data set of manually segmented images, which was used to develop the deep learning semantic segmentation approach. These image processing operations were performed using the plug im! Software.^35^

### SEM Image Processing for Porosity Quantification

#### Mean Porosity from SEM Images

**Figure 4:**
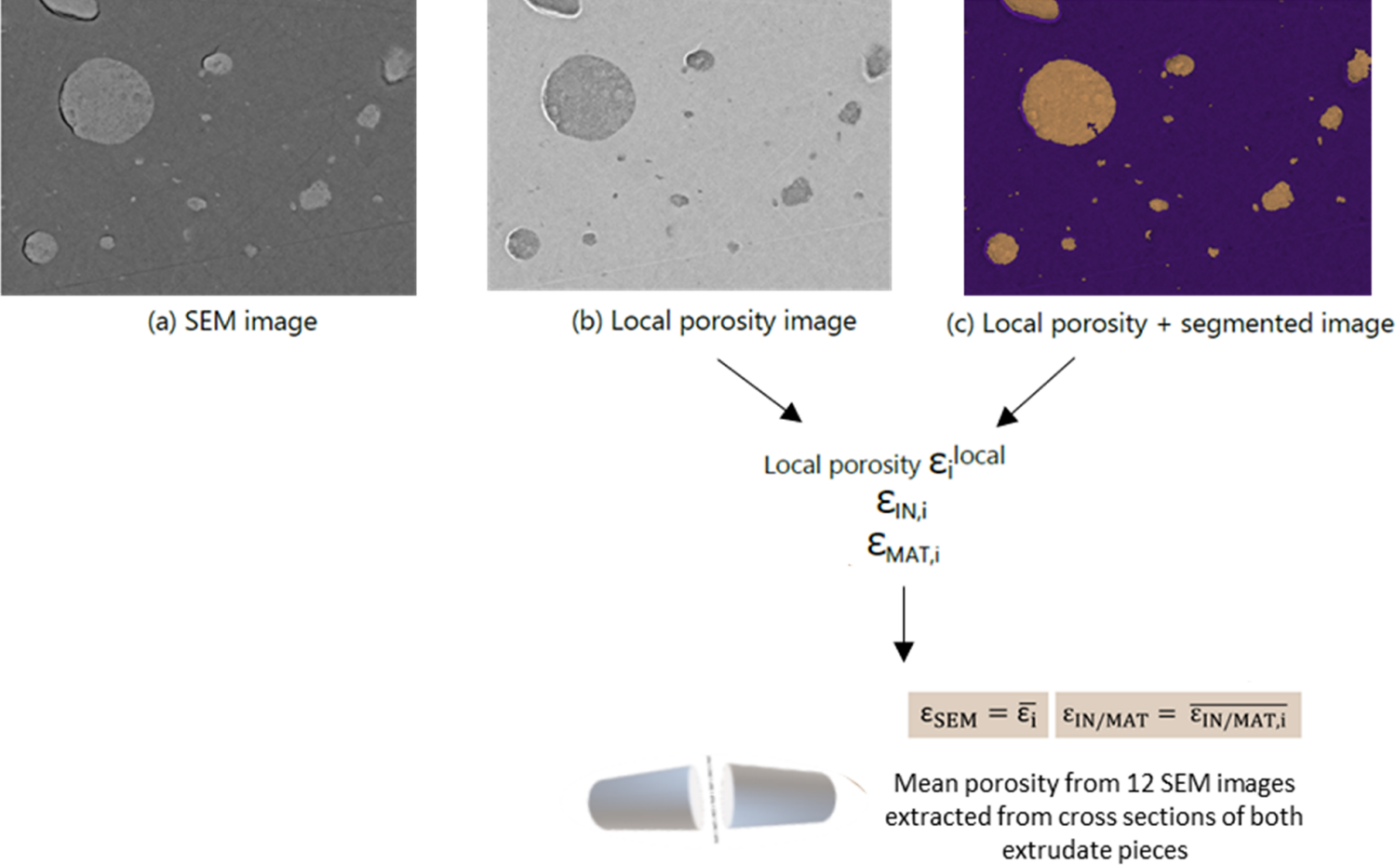
SEM imaging and processing methodology for porosity quantification: (a) SEM image acquired on predefined zone of alumina B, (b) local porosity image obtained using gray-level calibration method to evaluate ε_i_^local^, (c) segmented image applied to local porosity image to extract inclusion and matrix porosity. Extrudate mean ε_SEM_, inclusion and matrix porosity ε_*I*N/MAT_ calculated based on a total number of 12 images acquired for cross sections of both extrudate pieces/sections.

To measure the local porosity from SEM images acquired in the backscattered electron mode (example in Figure 4a), the porosity needs to be determined in each pixel of an image. This becomes possible through adequate sample preparation based on the impregnation of the porous material with a resin, which was applied in this study.

If we assume that the prepared γ-alumina samples represent a homogeneous mixture of the solid bulk alumina phase and the resin, the local porosity for an SEM image becomes directly related to the local resin concentration in the pores. Therefore, local porosity determination from SEM images requires the use of two reference materials to calibrate the image gray levels, in this case, α-alumina and the PMMA resin. SEM images acquired for these materials, as described by Sorbier et al.,^11^ were used to calibrate the gray levels corresponding to a porosity of 0 and 1, respectively. For calibration of zero-porosity, α-alumina in the form of a sapphire sphere had to be used instead of porous γ-alumina, since the latter contains structural defects leading to a nonuniform level of gray, which does not allow correct gray-level calibration for porosity evaluation.

After calibration of the two extreme porosity values with reference materials, the local porosity ε_i_^local^ for each SEM image was determined by using a calibration model proposed by Sorbier et al.,^11^ based on Donovan’s electron fraction mixing rule^36^
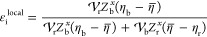
3where  and  are the molar volumes of the bulk alumina phase and the PMMA resin, respectively, *Z*_b_ and *Z*_r_ the modified atomic numbers (Table S1, see Supporting Information), η_b_ and η_r_ are the backscattering coefficients of the two materials, and η̅ is the backscattering coefficient measured for the SEM image. In Donovan’s relation, the empirical exponent *x* of 1.4 is used, whereas Sorbier et al. proposed *x* = 1.319.^11^ We have used a corrected empirical exponent of *x* = 0.865 obtained through least-squares fitting on Monte Carlo simulation results obtained for homogeneous virtual alumina samples that lead to excellent agreement over the whole porosity range. Application of eq 3 allowed porosity calculations in this study based on known _b_, _r_, *Z*_b_, *Z*_r_, and η_b_, η_r_, η̅ measured by SEM. An example of a local porosity image resulting from this approach is shown in Figure 4b.

The mean porosity of each support ε_SEM_ was evaluated based on local porosities ε_i_^local^ obtained for the entire SEM image set of *m* = 12 images acquired on predefined zones of both extrudate sections, and the standard deviation was calculated for the whole image set.

#### Alumina Inclusion and Matrix Porosity from Segmented SEM Images

For each alumina support, the entire SEM image set acquired for mean porosity evaluation was processed by using the segmentation strategies described earlier, which enabled the segmentation of local porosity images into two classes representing the alumina heterogeneities and the alumina matrix. By coupling the local porosity image (Figure 4b) with the segmented binary image, a 2D map of the image porosity (Figure 4c) is obtained and employed to selectively determine the porosity of each phase using eq 3. The mean porosity of alumina heterogeneities ε_IN_ and matrix ε_MAT_ was evaluated based on the entire image set acquired for each sample, and the standard deviation was calculated.

### Impact of Spatial Heterogeneity on Mass Transfer

Local quantification results obtained for the alumina heterogeneities and matrix by SEM image processing were used as input for two-phase analytical models,^24^ originally derived for predictions of effective electrical conductivity,^25,27^ to predict the contribution of both phases on the effective tortuosity factor τ_eff_. Here, the effective tortuosity factor is strictly related to the diffusion of molecules in the porous network and describes the extent to which the diffusion path of molecules in the porous medium increases with respect to their molecular diffusion path in the liquid phase for an equivalent mean square displacement. For its prediction, basic two-phase models were chosen so as to best represent the actual form and distribution of alumina heterogeneities (i.e., inclusions) in a continuous matrix.

The first model used was the Bruggeman-type effective medium theory (EMT) model,^25^ which applies to two-phase materials with a random distribution of both components. For spherical inclusions with a tortuosity factor τ_IN_ and volume fraction ϕ embedded in a matrix with a tortuosity factor τ_MAT_ and volume fraction 1 – ϕ, the model takes the form
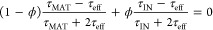
4

The second model used for comparison was the reciprocity model derived by del Río et al.,^27^ based on Keller’s reciprocity theorem^26^ with the assumption that a two-component microstructure remains statistically equivalent when interchanging the component volume fractions. Also, this model has been established for a particular case of two-phase materials for which a characteristic inclusion shape cannot be defined and, therefore, is applicable to any material with a random distribution of inclusion shape that is isotropic in two dimensions. The reciprocity formula adapted to the case of effective tortuosity factor prediction is expressed as
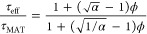
5where α is the local tortuosity factor ratio defined as
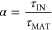
6

Furthermore, Gao and Gu^37^ confirmed the validity of the reciprocity model not only in two dimensions but also for three-dimensional materials by deriving the same equation from the Maxwell–Garnett-type approximation when considering shape distribution effects.

Without further information, the local tortuosity factors of alumina heterogeneities and matrix were estimated by using a theoretical tortuosity–porosity correlation proposed by Wakao and Smith^38^ based on porosities extracted independently for the two components via SEM image processing. The correlation takes the form of
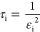
7where ε_i_ represents the alumina inclusion or alumina matrix porosity obtained from SEM image processing (i = IN or MAT). While other relationships could be used, this correlation was chosen to ensure the largest variation of tortuosity with porosity (Figure S2, see the Supporting Information), since this will allow the emphasis of the impact of inclusions on the effective tortuosity factor in the case of a sufficiently large difference between the porosities of both phases.

The reciprocity model was then applied to perform a sensitivity analysis to examine the variability of the effective tortuosity factor with the inclusion volume fraction and the porosity difference between both components.

## Results and Discussion

### Textural Properties

**Figure 5:**
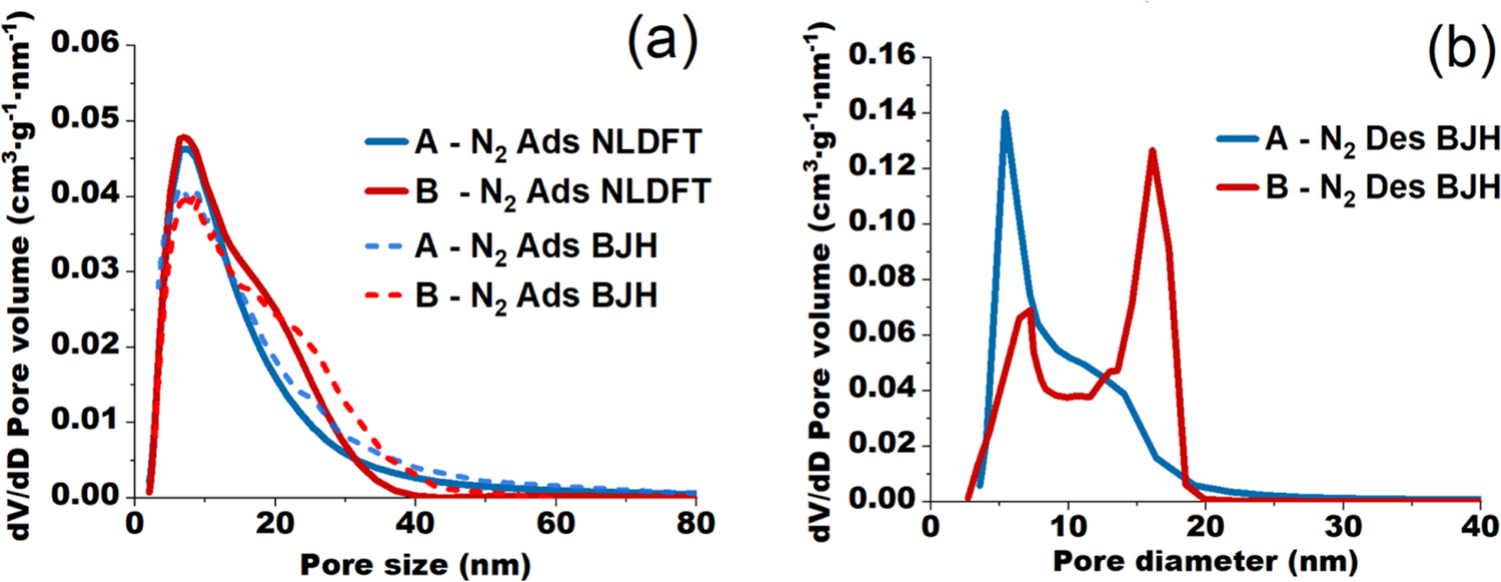
PSDs of studied γ-aluminas obtained by applying (a) the NLDFT model for slit pores and the BJH method to the N_2_ adsorption isotherm branch, and (b) the BJH method to the desorption branch.

The textural properties of the studied γ-aluminas, reported in Table 1, appear at first sight very similar for both supports. There is no variation between the total porosity values evaluated from MIP and N_2_ adsorption using ρ_s_ from helium pycnometry, which suggests that the supports are purely mesoporous. This is further evidenced by the lack of macropores in the mercury intrusion PSD (Figure S3, see the Supporting Information). Figure 5a presents the PSDs obtained for both supports by applying the NLDFT model for slit pores and the BJH method to the adsorption isotherm branch. For each alumina, only a slight difference is observed between the adsorption PSDs from BJH and NLDFT. These PSDs were found to be very close and centered at around 7 nm, implying a similar pore size range for the two aluminas. However, their PSDs obtained by the BJH method, shown in Figure 5b, vary significantly. For both supports, the desorption PSD shows a peak around 5–6 nm, but, for support A, a much larger volume is ascribed to these small mesopores. This could indicate a higher porous volume blocked by small mesoporous necks,^39−41^ and thus, more severe pore blocking effects for this support. This peak can alternatively be attributed to cavitation effects, indicating the presence of ink-bottle-shaped pores with small pore mouths. In both cases, it can be concluded that the pore network geometries of the two samples are different, inducing potential impact on mass transfer^42−44^ and different diffusional properties for both aluminas.

**Table 1:** Textural Properties of the Studied γ-Alumina Supports

alumina support	*S*_BET_ (m^2^·g^–1^)	ρ_g_ (g·cm^–3^)	ρ_s_ (g·cm^–3^)	ε_MIP_	*V*_pore_ (cm^3^·g^–1^)	ε_N_2__
A	292 ± 29	0.89 ± 0.01	3.17 ± 0.01	0.72 ± 0.01	0.82 ± 0.01	0.72 ± 0.02
B	299 ± 30	0.91 ± 0.01	3.21 ± 0.01	0.72 ± 0.01	0.86 ± 0.01	0.73 ± 0.02

### Spatial Heterogeneity of γ-Alumina from SEM Imaging

**Figure 6:**
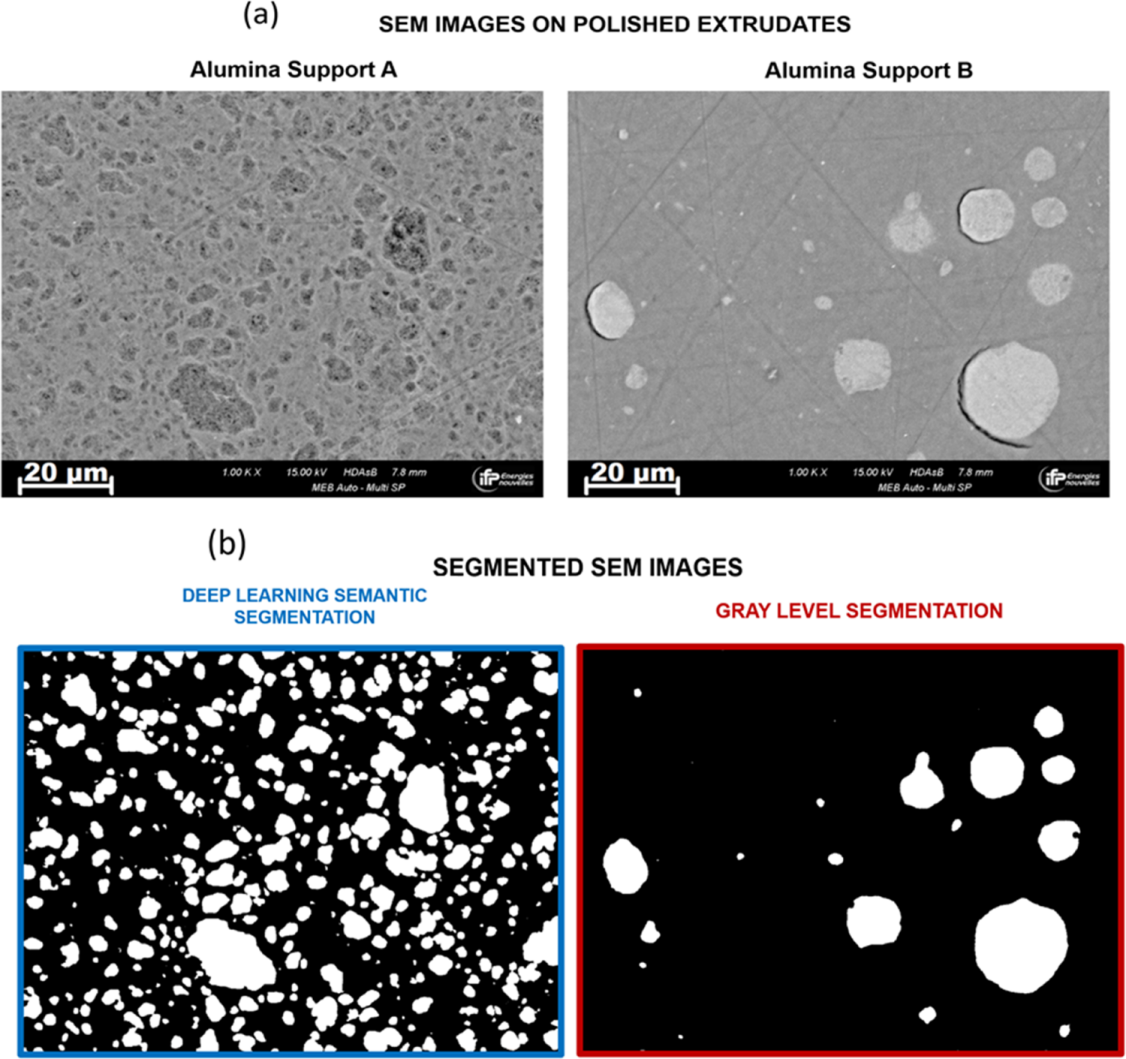
(a) Original SEM images on polished sections of alumina A and B embedded in PMMA resin and (b) the corresponding segmented images obtained via image processing.

SEM images of aluminas A and B performed on polished sections of extrudates embedded in the PMMA resin at 1000× magnification are shown in Figure 6a. For both samples, the images reveal the presence of alumina heterogeneities or inclusions of different sizes embedded in an alumina matrix, yet the density of these heterogeneities varies depending on support type. The alumina inclusions in support A are less dense, and, thus, more porous with respect to the surrounding alumina matrix, whereas support B contains heterogeneities that are denser compared to the matrix. This implies that the porosity is higher inside the alumina heterogeneities of support A and the alumina matrix of support B. However, the variation between inclusion and matrix porosity appears to be more significant for sample B when considering the gray-level difference. The formation of two γ-alumina phases of different density is ascribed to partial dispersion (peptization) of boehmite aggregates during the shaping procedure^6^ and implies a variation in the organization of elementary alumina nanoparticles between the alumina heterogeneities and the matrix.

The presence of a second γ-alumina phase in the form of inclusions having a different porosity with respect to the surrounding continuous alumina matrix could have an important impact on the effective diffusivity obtained from the contributions of both phases. Whether this impact is significant will strongly depend on the porosity difference between the alumina matrix and the heterogeneities, and on the volume fraction occupied by these inclusions.^24^ Therefore, a complex quantification of these properties is essential to assess the effect of heterogeneities on mass transfer. To enable such quantification, an adequate semantic segmentation strategy was selected depending on the support type and employed to perform SEM image binarization. The results obtained for both aluminas are presented in Figure 6b. It is clear that the use of segmentation strategies individually adapted for each support type is crucial, as this allowed us to obtain binarized images that correspond very well to those from SEM, with alumina inclusions precisely distinguished from the matrix to facilitate their quantification, and thus mass transfer predictions.

### Total Alumina Inclusion Surface Fraction and Spatial Distribution

Table 2 reports the total surface fraction θ of alumina heterogeneities determined for the whole cross sections of both extrudate pieces of each support and the averaged values for the entire extrudate θ̅. As predicted from qualitative analysis of SEM images, the quantification revealed a higher total average surface fraction of alumina inclusions θ̅ of around 30% for support extrudate A with respect to extrudate B, where inclusions occupy only 10% of the pellet cross section. The total surface fraction of alumina heterogeneities does not vary between the cross sections of both extrudate pieces for each support. This suggests that the surface probed by imaging is large enough compared to the representative volume element (RVE) for quantifying this surface fraction. For isotropic media, basic stereology ensures that this surface fraction is equal to the volume fraction.

**Table 2:** Total Inclusion Surface Fraction for Each Extrudate Section θ and the Average Values Calculated for the Entire Extrudate θ̅

	total inclusion surface fraction **θ**		
alumina support	1st extrudate section	2nd extrudate section	θ̅
A	0.306 ± 0.025	0.300 ± 0.022	0.303 ± 0.023
B	0.109 ± 0.065	0.093 ± 0.084	0.101 ± 0.075

**Figure 7:**
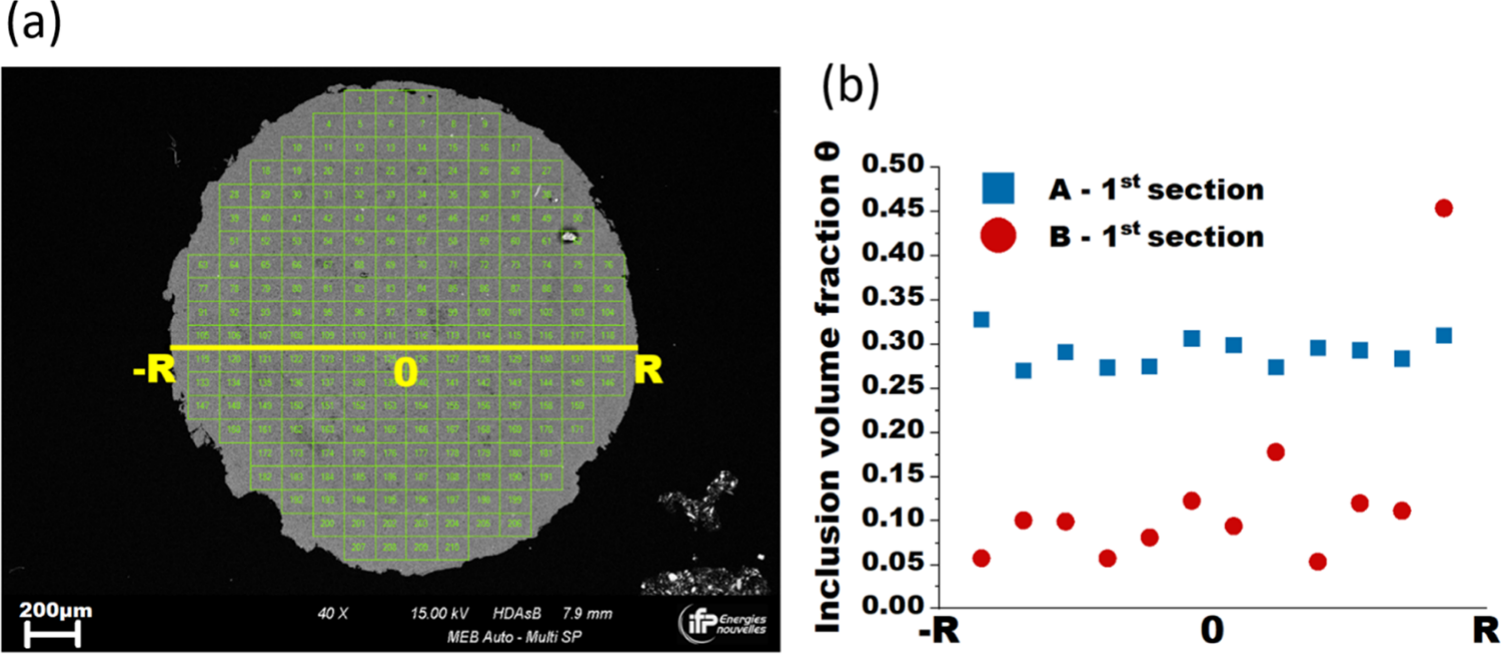
(a) SEM image of alumina A extrudate cross section digitally divided into individual images of 1024 × 768 pixels and (b) spatial distribution of inclusion surface/volume fraction across the entire diameter of the first extrudate section for supports A and B.

Interestingly, the standard deviation of inclusion surface/volume fraction, calculated based on the entire image set obtained for the whole cross section, is higher for both extrudate pieces of support B. This indicates a larger spatial variation of inclusion volume fraction between different zones of extrudate B. The spatial distribution of inclusion volume fraction was further examined for one row of SEM images acquired across the entire diameter of one of the extrudate sections, as illustrated in Figure 7a, and the results obtained for both aluminas are presented in Figure 7b. Table 3 reports the average inclusion surface/volume fractions for data points plotted in Figure 7b with calculated standard deviations, confirming a larger variation of alumina heterogeneity volume fraction of ±11% across the extrudate diameter for support B, which suggests a more heterogeneous distribution of inclusions within the support with respect to alumina A. For the SEM image acquired at point *R* of the support B’s cross section, an inclusion volume fraction of over 45% was obtained, which is ascribed to the presence of a very large alumina inclusion agglomerate (>100 μm) and not linked to boundary effects.

**Table 3:** Average Inclusion Surface Fraction across Extrudate Diameter θ̅_d_ with Standard Deviation Calculated for Data Points in Figure 7b

	average inclusion surface fraction across extrudate diameter **θ̅**_**d**_	
alumina support	θ̅_d_	standard deviation
A	0.29	0.02
B	0.13	0.11

### Alumina Inclusion Size

**Figure 8:**
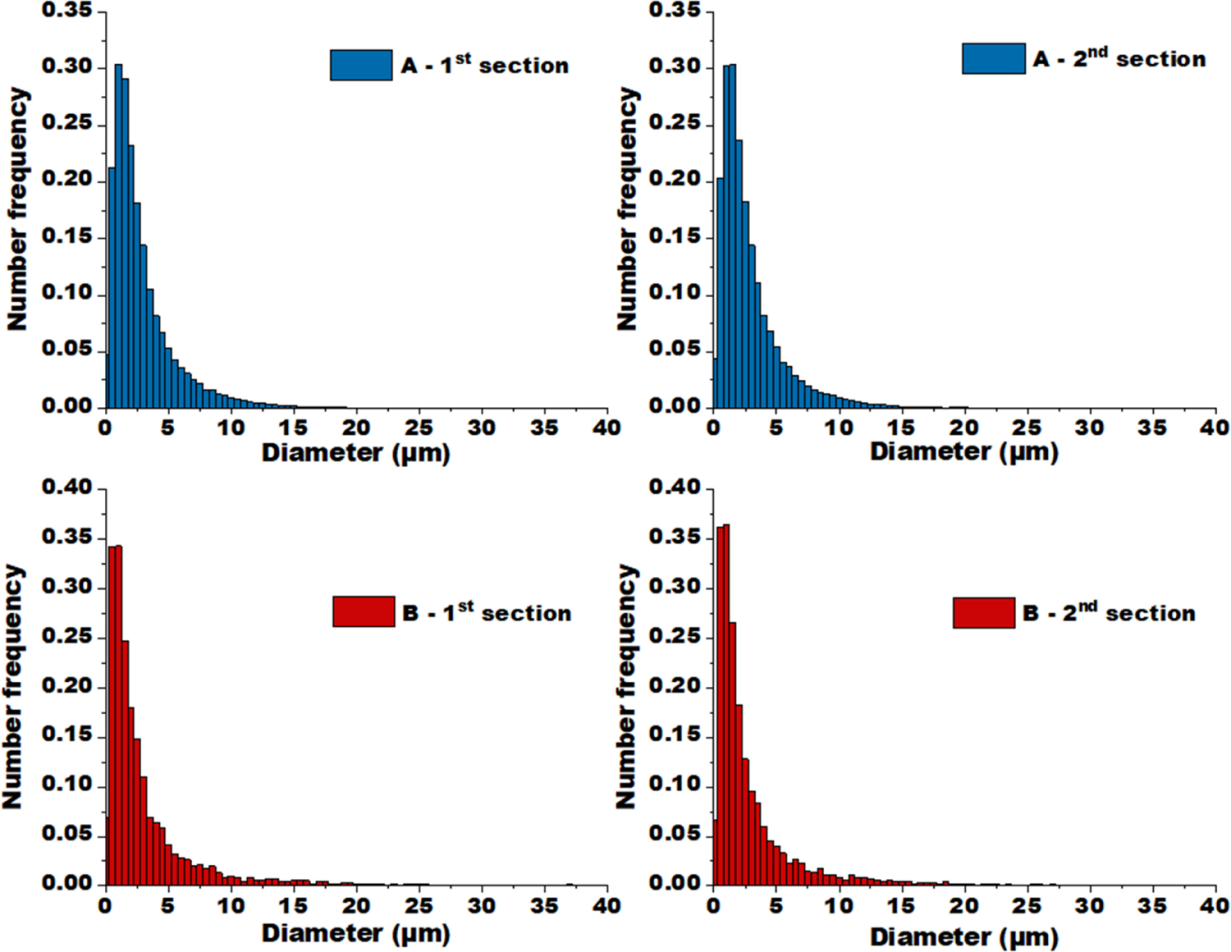
Number frequency histograms of alumina inclusion diameter for both sections of extrudates of supports A and B.

Figure 8 presents number frequency histograms of alumina inclusion diameters obtained for both sections of extrudates A and B based on the entire image set. For each extrudate piece, the total number of heterogeneities in the cross section and the average inclusion diameter *d* were calculated, and the results are reported in Table 4. The histograms reveal a similar inclusion size distribution for both supports ranging up to around 20 μm for alumina A and 25 μm for alumina B, and overall extrudate average inclusion diameters *d̅* > 3 μm in both cases. This implies that the two studied aluminas are comparable as far as the inclusion size is concerned, yet the quantity of alumina heterogeneities is around 10 times higher for support A. This is consistent with surface/volume fraction quantification results revealing a larger contribution of alumina inclusions to the total extrudate volume for this sample.

**Table 4:** Total Number of SEM Images and Inclusions for Both Extrudate Sections, and the Extrudate Average Inclusion Diameter *d̅* Calculated Based on the Combined Image Set

alumina support	number of SEM images ***n***	number of inclusions	***d*** (μm)	standard deviation (μm)	***d̅*** (μm)	standard deviation (μm)
A—1st section	160	49 447	3.16	2.79		
					3.14	2.75
A—2nd section	146	47 146	3.12	2.70		
B—1st section	144	4 920	3.50	4.42		
					3.41	4.29
B—2nd section	133	4 370	3.31	4.16		

### Mean Porosity from SEM Images

Table 5 reports the mean porosity values from SEM, ε_SEM_, and the standard deviation for the studied samples, computed based on local porosities measured for the entire SEM image set of *m* = 12 images. Overall, a slightly higher mean porosity was obtained for alumina A exceeding that of support B by only 2.3%. The mean porosities were then compared with mercury intrusion porosity values ε_MIP_, reported in Table 1, and a very good agreement was found for both aluminas, with an absolute relative error of 4% and below 6% for supports B and A, respectively, thereby confirming the reliability and accuracy of the mean porosity measurement by SEM.

**Table 5:** Mean Porosity from SEM Images, ε_SEM_, Calculated Based on a Set of 12 Images Acquired on Predefined Zones of Both Extrudate Sections of Aluminas A and B with Standard Deviation

alumina support	**ε_SEM_**	standard deviation
A	0.764	0.005
B	0.747	0.012

### Alumina Inclusion and Matrix Porosity

Table 6 reports the average alumina inclusion and matrix porosities (ε_IN_ and ε_MAT_, respectively) obtained by segmentation of the SEM image set acquired for the evaluation of mean porosity. The results reveal that the matrix porosity for both aluminas is nearly identical, differing by only 1.3%. Furthermore, matrix porosities are comparable to the mean porosity values from SEM. This seems reasonable, since for support A, despite a 30% inclusion volume fraction, the porosity difference between the inclusions and matrix is only 7%. In the case of support B, alumina inclusions constitute only 10% of the extrudate volume, and, therefore, their presence has insignificant impact on the mean porosity measured by SEM. However, a much larger variation of over 21% was found between matrix and inclusion porosity for this support, which could, on the other hand, have a remarkable effect on the effective tortuosity factor resulting from the contribution of both phases.

**Table 6:** Alumina Inclusion and Matrix Porosities from SEM Images with Standard Deviation

	support			
porosity	alumina A	standard deviation	alumina B	standard deviation
ε_IN_	0.803	0.006	0.626	0.013
ε_MAT_	0.752	0.005	0.762	0.008

### Prediction of Spatial Heterogeneity Impact on Mass Transfer Using Local SEM Quantification Results

Table 7 reports local alumina matrix and inclusion tortuosity factors (τ_MAT_ and τ_IN_, respectively) calculated using eq 7 based on porosity values measured selectively by SEM for both phases. The Wakao and Smith correlation^38^ gives a nearly 50% difference between the inclusion and matrix tortuosity factor for support B due to a much larger interphase porosity variation compared to support A for which, consequently, the tortuosity factor difference is only around 14%. These local tortuosity factors and the inclusion volume fractions obtained via image processing served as input for EMT and reciprocity models to predict the effective tortuosity factor τ_eff_ from the contribution of both phases, reported in Table 7. For each support, both models give the same τ_eff_, despite a different assumption regarding the inclusion shape (i.e., spherical for the EMT model and no particular inclusion shape for the reciprocity model). Consequently, the same ratio of the predicted effective tortuosity factor to matrix tortuosity factor  is obtained. Interestingly, for the two aluminas, this ratio is close to unity, which implies that the effective tortuosity factor is comparable to that of the matrix and allows us to conclude that the impact of spatial heterogeneity in the form of alumina inclusions of different density on the effective tortuosity factor is insignificant for both studied supports. For alumina A, this is due to a very small interphase porosity variation (i.e., 7% difference), despite a relatively large inclusion volume fraction of 30%, whereas for support B, the porosity difference is remarkable (i.e., over 21%) yet the inclusion content is insufficient to profoundly influence τ_eff_.

**Table 7:** Local Tortuosity Factors Calculated Using Equation 7 from Matrix and Inclusion Porosities Measured by SEM (α = τ_IN_/τ_MAT_); Effective Tortuosity Factor Predicted Using EMT and Reciprocity Models and Its Ratio to τ_MAT_

	local tortuosity factor **τ**_**i**_ (eq 7)			EMT model (eq 4)		reciprocity model (eq 5)	
alumina support	τ_MAT_	τ_IN_	α	τ_eff_		τ_eff_	
A	1.77	1.55	0.88	1.70	0.96	1.70	0.96
B	1.72	2.55	1.48	1.80	1.04	1.79	1.04

**Figure 9:**
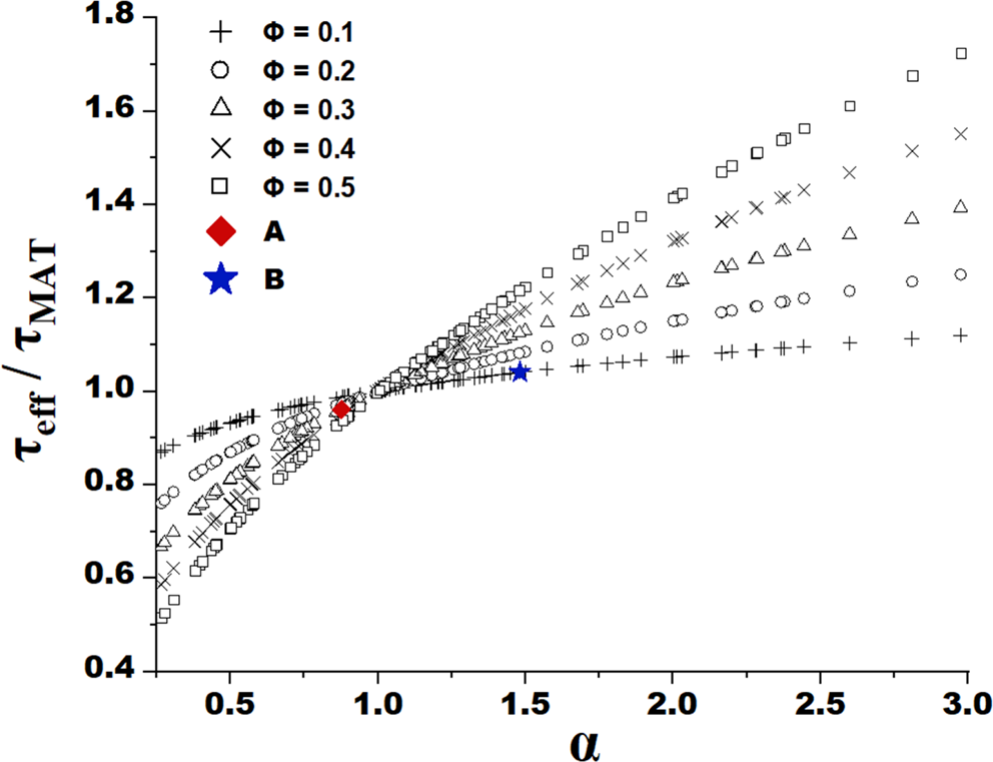
Ratio of effective tortuosity factor τ_eff_ predicted by the reciprocity model to matrix tortuosity factor calculated using eq 5, plotted as a function of local tortuosity factor ratio α = τ_IN_/τ_MAT_. Here, ϕ is the volume fraction of the inclusions embedded in the matrix, and A and B indicate the two studied alumina supports.

Sensitivity analysis was conducted to determine the interphase porosity variation and inclusion volume fraction ϕ, for which the effect of spatial heterogeneity on τ_eff_, predicted by the reciprocity model, becomes non-negligible. Figure 9 shows the variation of  as a function of the local tortuosity factor ratio  for different inclusion content ϕ ranging from 10 to 50%. Crystallites of boehmite, being the precursor of γ-alumina, are morphologically close to cylinders, and thus, according to Zou and Yu,^45^ a stacking of cylindrical boehmite particles with a length-to-diameter ratio between 2 and 4 leads to porosities between 0.4 and 0.45. Keeping this in mind and considering that the maximal porosity measured for inclusions of alumina A is 80%, the porosity was varied between 0.4 and 0.8 for calculations. The results show that to see a profound impact of dense alumina inclusions of support B on τ_eff_, with an over 21% porosity variation with respect to the matrix corresponding to an α of 1.48, the inclusion volume fraction would need to be of over 30%. In the case of alumina A with more porous inclusions compared to the matrix, the interphase porosity difference of 7% yielding an α of 0.88 is insufficient to induce an important influence of these porous heterogeneities on τ_eff_, even if their volume fraction increases to 50% or more. However, their impact becomes non-negligible for a 30% volume fraction measured by SEM, when α approaches 0.66, which corresponds to a porosity variation of around 23%. Overall, it can be concluded that the impact of spatial heterogeneity on effective diffusivity significantly rises with the increase of interphase porosity difference and inclusion volume fraction.

## Conclusions

In this work, a specialized SEM imaging and sample preparation approach proposed by Sorbier et al.^11^ were employed to characterize γ-alumina spatial heterogeneity, which allowed us to represent its spatial organization in the form of alumina heterogeneities or inclusions of different density, depending on support type, and embedded in an alumina matrix. Local porosity measurements using Sorbier et al.’s^11^ method applied to SEM images led to mean porosity values that were in good agreement with values derived from mercury intrusion porosimetry, confirming SEM as a viable tool to evaluate porosity.

This study revealed that classical gray-level segmentation cannot be applied for γ-aluminas in general because of minor gray-level variations and profound textural complexity differences between the inclusions and matrix. This led to the development of a more sophisticated approach based on deep learning semantic segmentation, yielding binary images with alumina inclusions precisely distinguished from the matrix. This enabled accurate determination of inclusion size and volume fraction in the support, the latter being a key parameter for mass transfer predictions. By applying advanced, multistep SEM image segmentation on cross sections of the materials, local porosity could be consistently quantified, and a 2D porosity map was obtained, allowing to selectively extract the porosity of each phase (inclusions and matrix).

These local properties, provided exclusively by deep-learning-assisted SEM image analysis, enabled this first report of the impact of γ-alumina’s mesoporous spatial heterogeneity on mass transfer. For the studied supports, the interphase porosity difference and inclusion volume fraction were found to be insufficient to generate an important influence on the effective tortuosity factor, which was found to be close to that of the matrix in both cases. However, the study has shown that spatial heterogeneity impact becomes significant for an over 20% interphase porosity difference and inclusion volume fraction of around 30%. The effects of heterogeneity on mass transfer will be studied experimentally in an upcoming publication.

## Supporting Information Available

Physical properties of alumina and poly(methyl methacrylate) (PMMA) resin with uncertainties; plot of theoretical tortuosity–porosity correlations; mercury intrusion pore size distributions of studied γ-alumina supports; and grayscale level vs deep learning segmentation for sample A (PDF)
