# Supplementary material for: SEM Image Processing Assisted by Deep Learning to Quantify Mesoporous γ-Alumina Spatial Heterogeneity and Its Predicted Impact on Mass Transfer
Source: J Phys Chem C Nanomater Interfaces. 2024 May 13;128(20):8395–407. doi: 10.1021/acs.jpcc.4c00323 (PMC11129297; doi:10.1021/acs.jpcc.4c00323)
Supplement: Supplementary file 1 — jp4c00323_si_001.pdf [file jp4c00323_si_001.pdf]

SEM image processing assisted by deep learning to quantify mesoporous γ-alumina spatial heterogeneity and its predicted impact on mass transfer

Supporting Information

Aleksandra Głowska[†, §], *Elsa Jolimaitre*[*, §], *Adam Hammoumi*[§], *Maxime Moreaud*[§], *Loïc Sorbier*[§], *Caroline de Faria Barros*[§], *Veronique Lefebvre*[§], *Marc-Olivier Coppens*[*, †]

[†] Centre for Nature Inspired Engineering and Department of Chemical Engineering, University College London, London WC1E 7JE, United Kingdom

[§] IFP Energies nouvelles, Rond-point de l'échangeur de Solaize, BP 3, Solaize 69360, France

Corresponding authors:

Elsa Jolimaitre

E-mail: elsa.jolimaitre@sosponge.com;

ORCID : https://orcid.org/0000-0003-0962-1658

Marc-Olivier Coppens

E-mail: m.coppens@ucl.ac.uk

ORCID: https://orcid.org/0000-0002-1810-2537

Table S1.

Physical properties of alumina and poly(methyl methacrylate) (PMMA) resin with uncertainties. For PMMA, the density given is that of the polymer while the molar mass is that of the monomer.

	Formula	Density $\boldsymbol{\rho}$ (g·cm^{-3})	Molar mass $\boldsymbol{M}$ (g·mol^{-1})	Molar volume $\boldsymbol{\mathcal{V}}$ (cm^3·mol^{-1})	Modified atomic number $\boldsymbol{Z}$
Alumina	Al_2O_3	3.65±0.06	101.96±0.01	27.93±0.46	36.553±0.746
PMMA	$(C_5H_8O_2)_n$	1.18±0.01	100.12±0.01	84.85±0.72	43.667±0.592

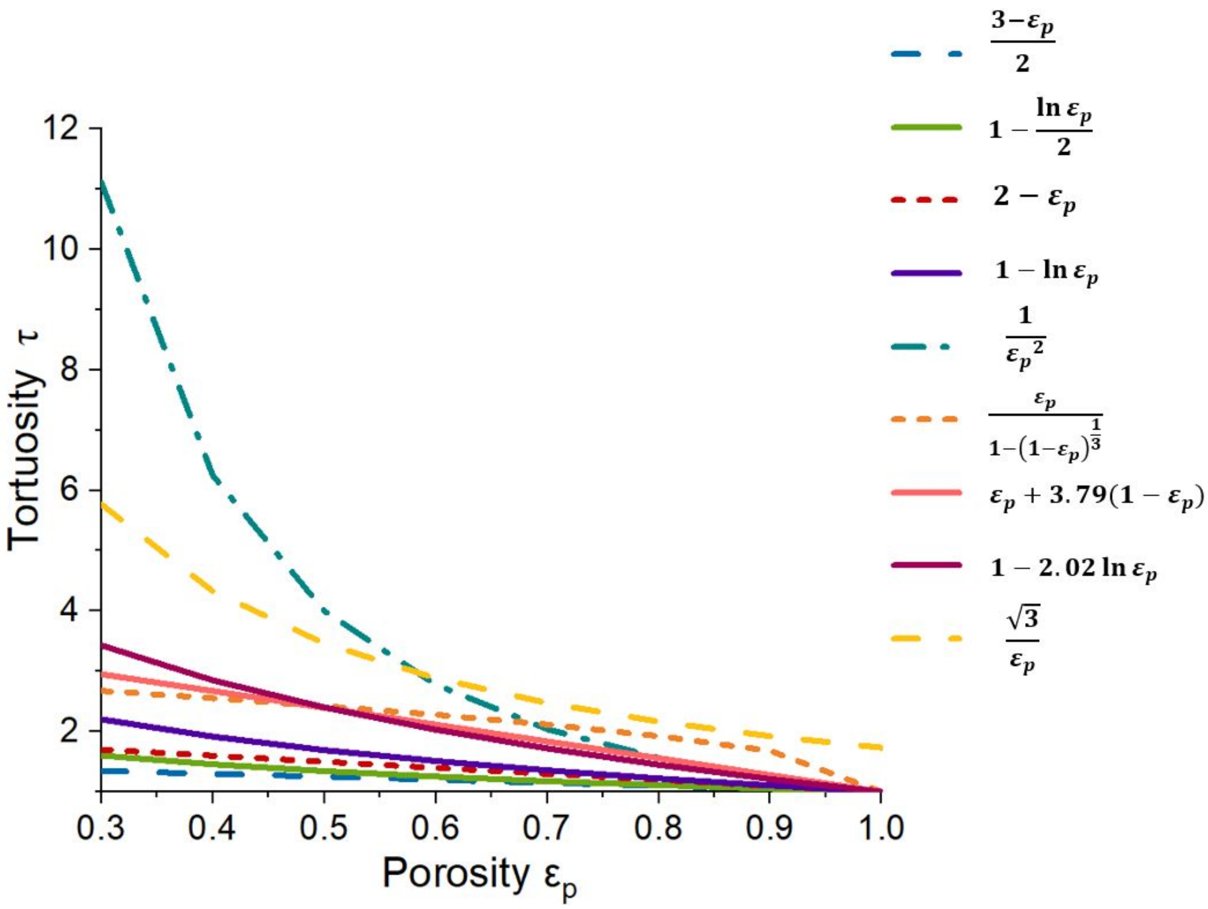

Figure S2. Plot of theoretical tortuosity-porosity correlations (Ref.: L. Shen, Z. Chen, Chemical Engineering Science 62 (2007) 3748–3755; B. Ghanbarian, A. Hunt, R. Ewing, M. Sahimi, Soil Science Society of America Journal 77 (2013) 1461).

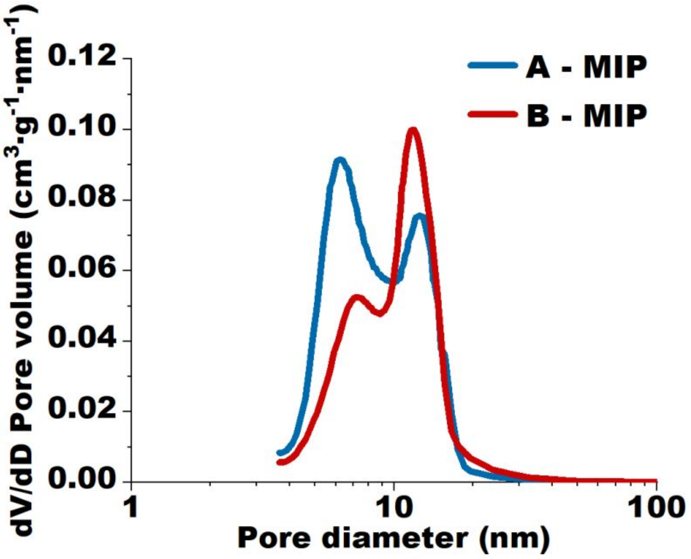

Figure S3. Mercury intrusion PSDs of studied γ-alumina supports.

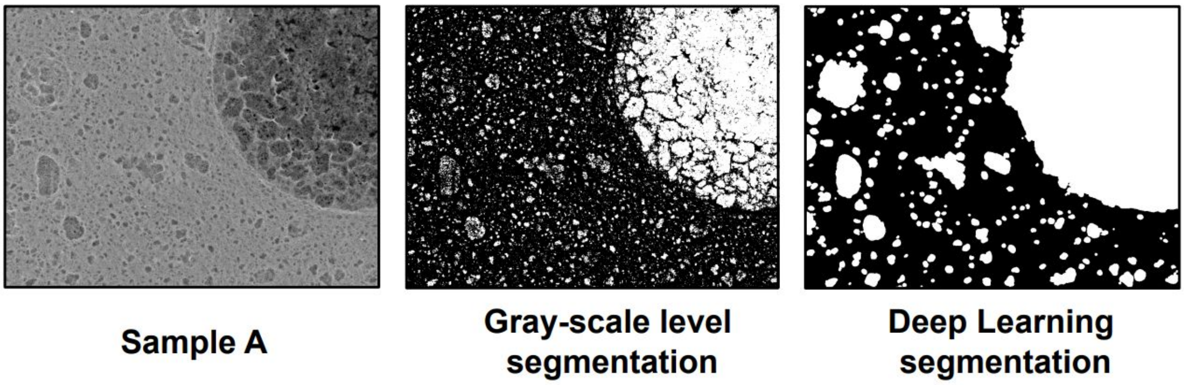

Figure S4. Gray-scale level vs. Deep Learning segmentation for sample A
